# Commentary: Constipation caused by anti-calcitonin gene-related peptide migraine therapeutics explained by antagonism of calcitonin gene-related peptide’s motor-stimulating and prosecretory function in the intestine

**DOI:** 10.3389/fphys.2022.1067274

**Published:** 2022-11-29

**Authors:** Kimberly D. Mackenzie, Mario Ortega, Yoel Kessler, Verena Ramirez Campos, Lynda J. Krasenbaum, Karen Carr, Xiaoping Ning, Jennifer Stratton

**Affiliations:** ^1^ Teva Pharmaceuticals, Redwood City, CA, United States; ^2^ Teva Branded Pharmaceutical Products R&D, Inc., West Chester, PA, United States; ^3^ Teva Pharmaceutical Industries Ltd., Tel Aviv, Israel

**Keywords:** fremanezumab, calcitonin gene-related peptide, CGRP, migraine, constipation

## Abstract

Calcitonin gene-related peptide (CGRP) pathway-targeted treatments have been shown to be efficacious in the prevention of episodic and chronic migraine. Currently approved therapies include monoclonal antibodies (mAbs) that target CGRP (eptinezumab, fremanezumab, and galcanezumab) and the CGRP receptor (erenumab), and small molecule CGRP receptor antagonists (atogepant and rimegepant). While CGRP pathway–targeted treatments are generally well-tolerated, in a review article by Holzer and Holzer-Petsche published in the January 2022 issue of *Frontiers in Physiology* the authors discussed the role of the CGRP pathway in gastrointestinal physiology, with a specific focus on constipation associated with the use of CGRP pathway–targeted treatments. The authors state that real-world surveys have shown constipation to be a “major adverse event” reported in “more than 50% of patients treated with erenumab, fremanezumab or galcanezumab.” As described in the current commentary, the limited data from the cited references in the review article by Holzer and Holzer-Petsche do not support that statement.

We read with interest the review article by Holzer and Holzer-Petsche published in the January issue of *Frontiers in Physiology*: “Constipation caused by anti-calcitonin gene-related peptide migraine therapeutics explained by antagonism of calcitonin gene-related peptide’s motor-stimulating and prosecretory function in the intestine*. Front Physiol*. 2022 January 11;12:820006.” In this review article, the authors discussed the role of the calcitonin gene-related peptide (CGRP) pathway in gastrointestinal (GI) physiology, with a specific focus on constipation associated with the use of CGRP pathway–targeted treatments for the prevention of migraine [the monoclonal antibody (mAb) against the CGRP receptor (erenumab); mAbs against the CGRP ligand (fremanezumab, galcanezumab)] and for the acute treatment of migraine [small molecule CGRP receptor antagonists (ubrogepant)] (Holzer and Holzer-Petsche, 2022). While we appreciate the authors’ intent to summarize investigations into CGRP pathway–targeted treatments and GI physiology, key methodological information was not described, and limited data were used to make broad conclusive statements linking constipation and the mAb therapies that target the CGRP pathway, including erenumab, fremanezumab, and galcanezumab.

Importantly, the authors did not disclose any methods for how studies and surveys were identified, specifically it is unclear if there were any predefined eligibility criteria for inclusion. As the authors noted, constipation rates from real-world studies and surveys were reported in patients with migraine, a history of multiple treatment failures, and concomitant drug use, including opioids, which have a well-established side effect of opioid-induced constipation (Müller-Lissner et al., 2017). While we acknowledge that the review by Holzer and Holzer-Petsche is narrative in nature, conclusions drawn from real-world data should be qualified when important patient characteristics (i.e., demographics, prior or concomitant treatments, and comorbidities) are not appropriately controlled for or balanced (Eichler et al., 2020).

Of note, Holzer and Holzer-Petsche (2022) state that post-approval, real-world surveys have shown that constipation is a “major adverse event” reported in “more than 50% of patients treated with erenumab, fremanezumab or galcanezumab.” However, the authors only present data from 1 study that shows a constipation rate above 50% (de Vries Lentsch et al., 2021; erenumab: 65%); all other cited studies show constipation rates below 50% (ubrogepant: 4.7%; erenumab: 7.6–43%; galcanezumab: 17.4–23%; fremanezumab: 23–25%) (Holzer and Holzer-Petsche, 2022). Moreover, the referenced data for fremanezumab are limited: the 25% constipation rate reported in the manuscript by Alex et al. (2020) was based on only a total of 4/16 patients, and the 23% constipation rate reported in Robbins and Phenicie (2020) was not specific to fremanezumab, but was based on combined data from 119 patients treated with erenumab, fremanezumab, or galcanezumab. Indeed, in a separate retrospective report by Robbins (2019), the constipation rate reported for patients taking fremanezumab (*n* = 79) was only 6%. While sparse, these real-world data on rates of constipation involving fremanezumab are well below the “more than 50%” constipation rate Holzer and Holzer-Petsche generalized to CGRP pathway–targeted treatments (Robbins, 2019; Robbins and Phenicie, 2020).

In summary, while we agree with the authors about the benefits of gathering real-world data on drug effectiveness and safety, the post-approval constipation rate data for fremanezumab presented by the Holzer and Holzer-Petsche are limited. It is inaccurate to make broad generalizations about adverse effects using those data. As additional real-world studies become available, a better understanding of the overall safety of these mAbs, including GI tolerability and constipation rates, may be possible.
